# Sexual Dimorphism in the Effects of Exercise on Metabolism of Lipids to Support Resting Metabolism

**DOI:** 10.3389/fendo.2014.00162

**Published:** 2014-10-07

**Authors:** Gregory C. Henderson

**Affiliations:** ^1^Department of Exercise Science, Rutgers Center for Lipid Research, Rutgers University, New Brunswick, NJ, USA

**Keywords:** post-exercise recovery, physical activity, fat oxidation, RMR, EPOC

## Abstract

Exercise training is generally a healthful activity and an effective intervention for reducing the risk of numerous chronic diseases including cardiovascular disease and diabetes. This is likely both a result of prevention of weight gain over time and direct effects of exercise on metabolism of lipids and the other macronutrient classes. Importantly, a single bout of exercise can alter lipid metabolism and metabolic rate for hours and even into the day following exercise, so individuals who regularly exercise, even if not performed every single day, overall could experience a substantial change in their resting metabolism that would reduce risk for metabolic diseases. However, resting metabolism does not respond similarly in all individuals to exercise participation, and indeed gender or sex is a major determinant of the response of resting lipid metabolism to prior exercise. In order to fully appreciate the metabolic effects and health benefits of exercise, the differences between men and women must be considered. In this article, the differences in the effects of exercise on resting metabolic rate, fuel selection after exercise, as well as the shuttling of triglyceride and fatty acids between tissues are discussed. Furthermore, concepts related to sex differences in the precision of homeostatic control and sex differences in the integration of metabolism between various organs are considered.

## Background

**Figure 1:**
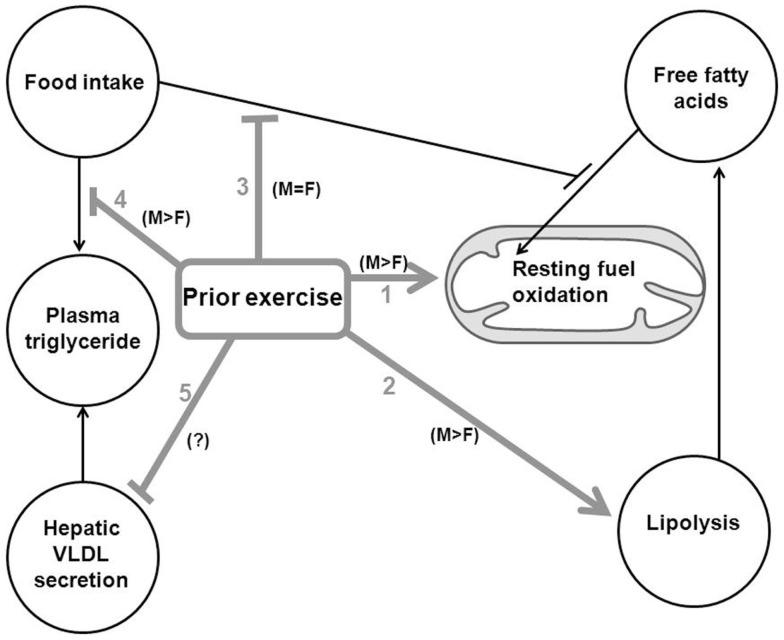
**Summary of the effects of an acute endurance exercise bout on subsequent metabolism of lipids in the support of the resting metabolic rate is shown**. M > F, response of males greater than that of females to a recent exercise bout. M = F, responses similar between males and females to a recent exercise bout. ?, Results for investigations of sex differences not yet reported. 1, recent exercise increases subsequent RMR (total substrate oxidation) in men but not significantly in women. 2, Exercise increases subsequent post-absorptive whole-body lipolysis in men but not in women. This higher lipolysis in men increases availability of FFA, which causes greater accentuation of post-absorptive fat oxidation in men than women. 3, Food intake generally inhibits subsequent fat oxidation, such that postprandial lipid oxidation is lower than post-absorptive lipid oxidation, but prior exercise blunts this inhibition of fat oxidation; thus, postprandial fat oxidation is enhanced by a recent exercise bout (similarly in men and women). 4, Food intake leads to transient elevation of plasma TG concentration (postprandial lipemia), but recent exercise blunts postprandial lipemia, likely to a greater extent in men than women. 5, Prior exercise blunts hepatic VLDL-TG secretion, but the sex difference is not yet clearly defined in the literature.

Chronic exercise training reduces all-cause mortality risk (1–4) and specifically shows a major beneficial impact on the risk for cardiovascular disease (CVD) (1–4) and diabetes (5–8). These risk-reducing effects in people who regularly exercise are likely a result of the prevention of future weight gain (9) as well as changes in lipid metabolism (10–14) and in metabolism of other nutrient classes such as carbohydrate (15–17). Many of the apparent benefits of chronic exercise participation may be a result of acute effects of the most recent exercise bout(s). For example, chronic exercise training increases resting fat oxidation (18), but even a single bout of exercise can lead to increased fat oxidation for hours or even on the following day (12, 19). Chronic exercise can also reduce hepatic triglyceride (TG) secretion or increase plasma TG clearance (20–23), but again, these results can be achieved even following a single exercise bout (14, 24, 25). It is critically important to understand physiological differences between populations in order to appreciate the complexity of physiology and responses to environmental stresses, and particularly it is clear that there are significant differences between men and women in response to exercise. Sex differences in the exercise response are exemplified by relatively greater reliance of women than men upon fat as an energy substrate during exercise (12, 26–35), and thus women are better able to spare carbohydrate and amino acids (36–38). Though still less explored than the responses during the exercise sessions, there are also numerous sex differences in metabolism during resting periods after exercise. Recent findings have described sexual dimorphism in substrate metabolism during the post-exercise recovery period and the role of lipid kinetics to support resting metabolism during this time period (Figure 1). Here, these aspects of sexual dimorphism after exercise are reviewed.

## Effects of Exercise on the Subsequent Resting Metabolic Rate

A single bout of exercise can lead to a modest but potentially significant elevation of the resting metabolic rate (RMR) for many hours afterward (9, 19, 39). This increase in RMR after exercise has been assessed through elevations in oxygen consumption (VO_2_)_._ Historically, the phenomenon of elevated VO_2_ after exercise had been referred to as oxygen debt, but the term “excess post-exercise oxygen consumption” (EPOC) was proposed as a more reasonable description of the phenomenon (39) and EPOC has now become a well-accepted term. Resting VO_2_ changes alone have been used by many investigators in attempts to study this phenomenon of altered RMR after exercise without consideration of the corresponding carbon dioxide production (VCO_2_). However, the caloric equivalence of VO_2_ depends upon relative fuel selection as indicated by the respiratory exchange ratio (RER) or as also referred to as the respiratory quotient (RQ). Indeed, the RER is altered after exercise (11, 12, 19), and thus the caloric equivalence of VO_2_ is altered (40). If the RER were 0.7, then the caloric equivalence of a liter of VO_2_ would be 4.7 kcal, while if the RER were 1.0, a liter of VO_2_ would correspond to an energy expenditure of 5.05 kcal (40). Thus, assessments of the acceleration of metabolism after exercise are flawed unless a true metabolic rate is calculated (e.g., in kilocalories/minute rather than simply in liters of oxygen). This variability in the metabolic energy equivalence of VO_2_ of slightly <10% is indeed modest, but certainly the EPOC phenomenon is very modest [e.g., an elevation of RMR of 0.1 kcal/min would be reasonably common (19)], so precision in assessment is essential. An additional methodological factor to consider is timing of assessments. In numerous studies of the EPOC phenomenon, post-exercise RMR (or simply VO_2_) was compared to pre-exercise values. However, RMR could drift throughout the day as a result of circadian changes in metabolism as well as reduced thermic effect of feeding (TEF) as time passes since the most recent meal. Furthermore, as the RER would drift downward across a day in the absence of multiple meals, then the caloric equivalence of VO_2_ would also decline across the day. Thus, it is important to conduct separate time-of-day matched sedentary control trials when attempting to perform a rigorous study of the effects of prior exercise on RMR.

Recently, we conducted a meta-analysis on post-exercise RMR (19). We expected that there could be sex differences in the degree to which RMR is accentuated by a recent exercise bout, so we were unable to interpret studies where men and women were combined into a single group in the analysis. For reasons explained above, we also only included studies in the analysis in which investigators performed time-of-day matched sedentary control trials. The results of this meta-analysis indicated that men experience a more robust increase in RMR than women after an endurance exercise bout (19). Furthermore, it appeared that the smaller increase in fat oxidation in women vs. men after exercise, discussed in the subsequent section of this article, is largely driven by this sex difference in the RMR response. It is not yet known why women exhibit less increase in RMR after exercise. However, this could be viewed as a higher level of precision in metabolic control in women than men, as discussed in greater detail later in this report. It appears that women are better able to resume normal resting metabolic parameters after exercise, whereas in men metabolism remains more significantly perturbed. It is possible that men experience a higher degree of respiratory uncoupling or a higher metabolic burden from processes such as lipolysis and gluconeogenesis after exercise. Indeed, as discussed below in detail, it is known that women reach resting rates of lipolysis (12) and hepatic glucose production (41) after exercise much more rapidly than men, and these observations correspond to the precise resumption of RMR after exercise (19).

## Effects of Exercise on the Subsequent Use of Lipid as a Fuel during Rest

Compared with rest, during exercise, the relative contribution of carbohydrate to fuel oxidation increases and the relative contribution of lipid decreases compared with rest (42–44). Thus, generally carbohydrate is the predominant fuel during exercise, especially if the intensity of exercise is vigorous (42–44), but after exercise there can be a shift toward lipid oxidation predominating in the support of the RMR for many hours (11, 12, 32, 45) and even into the next day (12, 14, 46, 47). As stores of glycogen are limited in the body, it could reasonably be expected that lipid oxidation would be elevated after exercise in proportion to the degree of glycogen depletion that occurred during exercise. After glycogen-depleting exercise, glycogen synthase activity is elevated in skeletal muscle (15), and this activation is associated with an accentuated lipid oxidation rate (48). This would likely be a result of the channeling of glucose toward storage, so it competes less with fatty acids (FAs) as a substrate for mitochondrial respiration.

During exercise, women rely more heavily upon lipid for fuel than do men, and thus women are better-equipped to spare carbohydrate (12, 26–35). Consistent with this finding of carbohydrate sparing in women during exercise, initial observations were that the increase in lipid oxidation after exercise was more pronounced in men than women when studied in the post-absorptive state (12, 32). Subsequently, a meta-analysis confirmed that in the post-absorptive state, the increase in lipid oxidation after endurance exercise was more robust in men than women (higher effect size in men) (19). However, this meta-analysis also indicated that the sex difference is abolished when men and women took a post-exercise meal and thus were in the postprandial state during assessments (19). Therefore, the sexual dimorphism is dependent upon nutritional status. The sex difference during exercise (higher reliance on carbohydrate in men) could theoretically explain the sex differences in fuel selection after exercise in men and women through effects of glycogen depletion on lipid oxidation. However, glycogen depletion actually does not appear to sufficiently predict patterns in post-exercise fuel selection, so it appears that other undiscovered cellular factors may be relevant. From a glycogen-centered viewpoint, one might predict that the higher carbohydrate use during exercise in men would lead to greater reduction in the RER after exercise in men than women, but a quantitative literature review (meta-analysis) indicated no sexual dimorphism in humans for the effect size of RER depression after exercise (19). Sex differences in post-exercise lipid oxidation appeared to be more closely related to the RMR than the RER. Further support for a glycogen-independent determinant of post-exercise lipid oxidation comes from the effects of nutritional state (postprandial vs. post-absorptive) on the sex difference in post-exercise lipid oxidation. The sex difference in post-exercise lipid oxidation is only present in the post-absorptive state (not in the postprandial state) (19). If the sex difference in lipid oxidation were the result of a need for glycogen replenishment, then one would expect a sizable difference in the postprandial state during net glycogen deposition. Thus, when searching for potential mechanisms for sex differences in post-exercise lipid oxidation, higher metabolic efficiency in women than men after exercise should be considered (19). Additionally, accentuation of lipolysis in men but not women after acute bouts of endurance exercise likely contributes to sex differences in post-exercise substrate oxidation though supply of FAs to β-oxidation (12). In summary, carbohydrate use during exercise might have some effect on post-exercise lipid oxidation, but the regulation of post-exercise substrate oxidation is far more complex, and the sexual dimorphism in post-exercise lipid oxidation is a result of factors that go beyond that of glycogen stores.

## Effects of Exercise on the Subsequent Shuttling of Triglyceride and Fatty Acids between Tissues

### Lipolysis and free fatty acid mobilization

In order to become available for inter-organ shuttling (e.g., from adipose tissue to muscle), the FAs from TG must be liberated by lipolysis. During complete lipolysis of a TG molecule, three FAs and one glycerol are released. However, despite this theoretical stoichiometry, the rate of appearance (Ra) of free fatty acid (FFA) in plasma remains lower than three times the glycerol Ra (12, 29, 30, 49–51). Therefore, FFA mobilization is less than the lipolytic rate, and this is believed to be a result of intracellular FA reesterification in adipose tissue, because this tissue can recycle FAs but cannot utilize free glycerol for TG synthesis *in vivo* (49). Glycerol Ra measures lipolysis but FFA Ra represents the true mobilization rate of FFA for distribution between tissues. These processes are measured by the use of stable isotope tracer methodology (12, 52). Glycerol and FFA mobilization are generally expected to follow similar patterns of change in response to stimuli, but FFA mobilization could also be effected by a change in intracellular metabolism of FA following lipolytic stimulation. In response to fasting for several days, lipolysis (53–55) and FFA mobilization (53–57) are increased. Lipolysis increases even over the duration of just a single day when meals are not consumed (58) and increases during exercise (12, 59, 60). Thus, it appears that lipolysis and related FFA mobilization are quite responsive to the energetic needs and fuel availability in the body. In men for hours after exercise, glycerol and FFA Ra remain substantially elevated above those of a sedentary control condition (12) and it was shown that men can exhibit this elevation even the day after exercise (61). However, the elevations of glycerol and FFA mobilization after exercise were substantially lower in women than men even after performing similar exercise sessions (12). These results for lipid mobilization, collected in the post-absorptive state, are believed to provide a mechanism for the lesser accentuation of lipid oxidation in women than men after exercise under these nutritional conditions through substrate supply to β-oxidation (12). The sex difference for resting lipolysis after exercise was most striking, as men exhibited approximately a 50% elevation for hours after endurance exercise, but women displayed absolutely no apparent elevation in lipolysis and instead very rapidly regained the resting lipolytic rate after exercise (12). This intensely homeostatic control of metabolism after exercise in women is discussed in more detail below under the section on homeostatic precision. Norepinephrine levels after exercise and the greater growth hormone response in men during exercise may have played a role in post-exercise sexual dimorphism in whole-body lipolysis, but these endocrine differences are not expected to be of an adequate magnitude to fully explain the sex difference in post-exercise lipolysis. Thus, while the predominant signal for post-exercise lipolytic control is not entirely clear, enhanced lipolysis in men is a likely explanation for sexual dimorphism in substrate oxidation in the post-absorptive state after exercise (12).

### Postprandial lipemia

Though plasma FFA are a major contributor to total fat oxidation in the post-absorptive state, in the fed state, the concentration of FFA in plasma drops while the availability of plasma TG increases, indicating a relative shift in the availability of different shuttling forms of FA (11). During the postprandial period, after taking a high-fat or even a mixed meal, this rise in the concentration of TG in circulation for hours is referred to as postprandial lipemia. During this period, plasma TG in the very low-density lipoprotein (VLDL) pool rises (hepatic TG secretion) in addition to that in the chylomicron pool (intestinal TG secretion) (46, 62), and FAs from the recent meal are rapidly recycled from initial appearance in chylomicrons into VLDL particles (63–65). In this process of postprandial TG shuttling, mild fluctuations in plasma TG concentration may be metabolically appropriate, but excessive postprandial lipemia increases risk of CVD (66–70); thus, regulation of the postprandial plasma TG excursion is important for health. A single bout of exercise, immediately before or even a day before a meal can profoundly blunt the response of postprandial lipemia (11, 13, 14, 46, 71–84). However, the excursion of postprandial plasma TG concentration (i.e., postprandial lipemia) is drastically lower in premenopausal women than men (64, 85–87), so the need to manage this aspect of metabolism is far lesser in young, lean women than men. This is a fundamental sexual dirmorphism in the need for physical activity to manage a metabolic parameter. However, because of the effect of obesity in exaggerating postprandial lipemia (88, 89), despite the very low plasma TG excursion in lean women, in obese women postprandial lipemia can be sizable (11), and in that case exercise can be quite efficacious in blunting the response to that which would be typically observed in a lean woman (11). Post-menopausal women also exhibit elevated postprandial lipemia (90), so ovarian hormones may be involved in the control of postprandial lipemia. In summary, exercise can blunt postprandial lipemia appreciably, but lean, premenopausal women are unique from men and unique from obese or post-menopausal women, in that they have very little room for improvement in postprandial lipemia. Young women would likely exhibit minimal capacity to benefit from a recent exercise bout for postprandial lipemia.

### Hepatic TG secretion

An additional aspect of TG shuttling through plasma is that of hepatic TG secretion, which contributes to postprandial lipemia (46, 62) but that is the sole source of plasma TG during the fasted state. Generally, this VLDL-TG secretion is studied in the post-absorptive state such that a steady state is present and such that chylomicrons do not contribute to plasma TG. It has been shown that VLDL-TG secretion rates are higher in women than men (91, 92), though it is not yet firmly established whether or not there is sexual dimorphism in the response to exercise. Chronic running wheel exercise vastly reduces the VLDL-TG secretion rate in rats (20, 21), so the rate of TG shuttling from the liver to other tissues appears to be modifiable by exercise. In men, VLDL-TG secretion rate was not reduced by a single recent bout of endurance exercise (14, 61), but in women, in a different study, a single session of a high volume of endurance exercise did indeed reduce subsequent resting VLDL-TG secretion (25). It is possible that there is sexual dimorphism in the response of resting VLDL-TG secretion to recent exercise, but this idea will need to be tested in a carefully controlled study in which the sexes are compared directly within a single study. Additionally, an animal model of this aspect of sexual dimorphism is needed in order to identify mechanisms, and this work is underway in our laboratory. In addition to the secretion rate of VLDL-TG from the liver, clearance of plasma TG can also be altered during the post-exercise recovery period (14, 25, 93–95); however, sexual dimorphism in the response of plasma TG clearance to exercise is not apparent. When exercise reduces hepatic TG secretion, the potential consequences of this reduction in TG export from the liver ought to be considered. Though this would reduce the supply of FAs to adipose tissue, which could be beneficial for managing the size of adipose depots, in the absence of any other changes, such as compensatory changes in FA uptake from plasma or changes in FA oxidation rates, then the reduced hepatic TG secretion would theoretically lead to an accumulation of hepatic TG. Thus, it is the balance of each of these processes that must be regulated, and likely appropriate compensation occurs eventually in response to reductions in hepatic TG secretion in healthy individuals. For example, increased hepatic mitochondrial density and capacity for FA oxidation in the liver were reported to be a response to chronic exercise training in rats (96–99), and this could provide compensatory FA disposal in response to the reduced VLDL secretion that has been observed under certain chronic exercise conditions (20–22).

## Precision of Homeostatic Control

In considering the variety of changes in resting metabolism to the stress of a recent exercise session, a pattern from the variety of sexual dimorphisms begins to emerge. Generally, women appear to be more precisely homeostatic than men. As discussed above, after exercise women rapidly regain euglycemia whereas men remain in a state of reduced blood glucose concentration for hours (41). Perhaps as part of a counter-regulatory response to the challenge to glycemia, men display a substantial elevation in lipolysis after exercise, while on the contrary, women quickly resume their normal resting rate of whole-body lipolysis (12). Furthermore, RMR is elevated significantly after exercise in men but to a negligible extent in women (19). The ability to spare energy expenditure and to retain rather than mobilize body fat stores would likely be a desirable trait for mammals, including humans, during the course of our evolutionary past. It is unclear why this trait of homeostatic precision has been of greater selective advantage in females than males, but one could speculate that metabolic precision is paramount in women because of the expected stresses on the body’s energy stores that are imposed by pregnancy and lactation. Furthermore, when considering other sex differences in homeostatic control, particularly those related to regulation of energy balance, it becomes apparent that female sex hormones play a role in the precision of homeostatic control of metabolic processes. For example, when challenged with a high-fat diet, female mice gain less body weight than male mice, but when the ovaries are surgically removed (ovariectomy), then this tight homeostatic control over energy balance is lost (100). It also appears that female rats (101–105) and possibly female humans (106, 107) are less prone to negative energy balance (weight loss or fat loss) when challenged with chronic exercise training, and indeed estrogen is known to generally act on neural control of behavior and metabolism for precise regulation of energy balance (108). Analogously, female rats also appear to demonstrate a better ability to cope with the metabolic stress of starvation compared with their male counterparts (109). Furthermore, in humans, even when controlling for the effect of age *per se*, there is an accelerated gain of weight and body fat after menopause (110–112), further implicating ovarian hormones in the control of energy metabolism. This concept of a sex difference in the precision of homeostatic control could provide an important context for past as well as future discoveries in the sexually dimorphic responses to single exercise bouts, chronic exercise training, and even sex differences in the tolerance of other physiological stressors. It appears that the tight regulation of lipid metabolism after exercise in women (Figure 1) fits into a general pattern in biology for sexual dimorphism in energy metabolism.

## Summary and Future Directions

The vast majority of the work on metabolic responses to exercise has addressed the changes in physiology and metabolism during the actual exercise bouts, but the majority of even an avid exerciser’s life is not spent exercising and rather is spent at rest. Thus, because of importance for developing a view of the overall impact of exercise on metabolism, the discoveries on resting metabolism in the post-exercise recovery period have been reviewed here. Indeed, continued work on this important aspect of exercise-related metabolism is needed to fully understand how exercise participation can change the integration of metabolism during the many hours of rest in the day. The majority of work on exercise has been conducted on males, so additional work on women and female laboratory animals is needed to further extend our understanding of sexual dimorphism in the future. Finally, in order to understand hormonal mechanisms, and relevance to post-menopausal women, additional work is needed on the postmenopausal human population and on ovariectomized (OVX) laboratory animals.

In summary, there are numerous changes in resting metabolism for hours or even a day after exercise (Figure 1). However, many of these changes in lipid metabolism and the metabolic energy demand are different between men and women after exercise. In isolation, each example of sexual dimorphism lacks a context. However, when viewed within the general pattern that emerges from the list of sex differences that have been reported, one can understand that females display a more precise defense of homeostasis during the post-exercise recovery period, including the control of the RMR, fasting lipolytic rate, postprandial TG concentration, blood glucose concentration, and fuel selection. The supply of lipid-based fuels to support mitochondrial respiration and to spare carbohydrate is depicted by a complex orchestration of flux of multiple metabolites between multiple tissues. This integration of lipolysis, FFA mobilization, lipoprotein kinetics, and fat oxidation with the RMR is clearly impacted by recent participation in an exercise bout, but generally to a lesser extent in women because of superior homeostatic control of metabolism and thus less perturbation of metabolism after exercise. In the future, it may be of benefit to discover ways to alter the response of resting metabolism to prior exercise, including the changes, which currently appear to be sex-dependent, in order to manipulate lipid metabolism in ways that will be ideal for the prevention of chronic disease as well as for the recovery from the energetic demands of exercise participation.

## Conflict of Interest Statement

The author declares that the research was conducted in the absence of any commercial or financial relationships that could be construed as a potential conflict of interest.

## References

[1] Blair SN Kohl HW III Barlow CE Paffenbarger RS Jr Gibbons LW Macera CA Changes in physical fitness and all-cause mortality. A prospective study of healthy and unhealthy men. JAMA (1995) 273:1093–8 10.1001/jama.1995.03520380029031 7707596

[2] Blair SN Kohl HW III Paffenbarger RS Jr Clark DG Cooper KH Gibbons LW Physical fitness and all-cause mortality. A prospective study of healthy men and women. JAMA (1989) 262:2395–401 10.1001/jama.1989.03430170057028 2795824

[3] Danaei G Ding EL Mozaffarian D Taylor B Rehm J Murray CJ The preventable causes of death in the United States: comparative risk assessment of dietary, lifestyle, and metabolic risk factors. PLoS Med (2009) 6:e1000058 10.1371/journal.pmed.1000058 19399161 PMC2667673

[4] Paffenbarger RS Jr Wing AL Hyde RT Physical activity as an index of heart attack risk in college alumni. Am J Epidemiol (1978) 108:161–75 707484 10.1093/oxfordjournals.aje.a112608

[5] Colberg SR Grieco CR Exercise in the treatment and prevention of diabetes. Curr Sports Med Rep (2009) 8:169–75 10.1249/JSR.0b013e3181ae0654 19584602

[6] Henriksen EJ Louters LL Stump CS Tipton CM Effects of prior exercise on the action of insulin-like growth factor I in skeletal muscle. Am J Physiol (1992) 263:E340–4 1514616 10.1152/ajpendo.1992.263.2.E340

[7] Helmrich SP Ragland DR Leung RW Paffenbarger RS Jr Physical activity and reduced occurrence of non-insulin-dependent diabetes mellitus. N Engl J Med (1991) 325:147–52 10.1056/NEJM199107183250302 2052059

[8] Ostergard T Andersen JL Nyholm B Lund S Nair KS Saltin B Impact of exercise training on insulin sensitivity, physical fitness, and muscle oxidative capacity in first-degree relatives of type 2 diabetic patients. Am J Physiol Endocrinol Metab (2006) 290:E998–1005 10.1152/ajpendo.00012.2005 16352678

[9] IOM. Dietary Reference Intakes for Energy, Carbohydrate, Fiber, Fat, Fatty Acids, Cholesterol, Protein, and Amino Acids. Washington, DC: National Academies Press (2002). 10.1016/s0002-8223(02)90346-9 12449285

[10] Schenk S Horowitz JF Acute exercise increases triglyceride synthesis in skeletal muscle and prevents fatty acid-induced insulin resistance. J Clin Invest (2007) 117:1690–8 10.1172/JCI30566 17510709 PMC1866251

[11] Davitt PM Arent SM Tuazon MA Golem DL Henderson GC Postprandial triglyceride and free fatty acid metabolism in obese women after either endurance or resistance exercise. J Appl Physiol (2013) 114:1743–54 10.1152/japplphysiol.00095.2013 23580597

[12] Henderson GC Fattor JA Horning MA Faghihnia N Johnson ML Mau TL Lipolysis and fatty acid metabolism in men and women during the postexercise recovery period. J Physiol (2007) 584:963–81 10.1113/jphysiol.2007.137331 17855762 PMC2277001

[13] Katsanos CS Grandjean PW Moffatt RJ Effects of low and moderate exercise intensity on postprandial lipemia and postheparin plasma lipoprotein lipase activity in physically active men. J Appl Physiol (1985) (2004) 96:181–8 10.1152/japplphysiol.00243.2003 12949025

[14] Magkos F Wright DC Patterson BW Mohammed BS Mittendorfer B Lipid metabolism response to a single, prolonged bout of endurance exercise in healthy young men. Am J Physiol Endocrinol Metab (2006) 290:E355–62 10.1152/ajpendo.00259.2005 16219668

[15] Bogardus C Thuillez P Ravussin E Vasquez B Narimiga M Azhar S Effect of muscle glycogen depletion on in vivo insulin action in man. J Clin Invest (1983) 72:1605–10 10.1172/JCI111119 6415114 PMC370448

[16] Lanza IR Short DK Short KR Raghavakaimal S Basu R Joyner MJ Endurance exercise as a countermeasure for aging. Diabetes (2008) 57:2933–42 10.2337/db08-0349 18716044 PMC2570389

[17] Ivy JL Kuo CH Regulation of GLUT4 protein and glycogen synthase during muscle glycogen synthesis after exercise. Acta Physiol Scand (1998) 162:295–304 10.1046/j.1365-201X.1998.0302e.x 9578375

[18] Romijn JA Klein S Coyle EF Sidossis LS Wolfe RR Strenuous endurance training increases lipolysis and triglyceride-fatty acid cycling at rest. J Appl Physiol (1993) 75:108–13 8376256 10.1152/jappl.1993.75.1.108

[19] Henderson GC Alderman BL Determinants of resting lipid oxidation in response to a prior bout of endurance exercise. J Appl Physiol (1985) 116(2014):95–103 10.1152/japplphysiol.00956.2013 24235102

[20] Simonelli C Eaton RP Reduced triglyceride secretion: a metabolic consequence of chronic exercise. Am J Physiol (1978) 234:E221–7 629336 10.1152/ajpendo.1978.234.3.E221

[21] Mondon CE Dolkas CB Tobey T Reaven GM Causes of the triglyceride-lowering effect of exercise training in rats. J Appl Physiol (1984) 57:1466–71 6394563 10.1152/jappl.1984.57.5.1466

[22] Tsekouras YE Magkos F Kellas Y Basioukas KN Kavouras SA Sidossis LS High-intensity interval aerobic training reduces hepatic very low-density lipoprotein-triglyceride secretion rate in men. Am J Physiol Endocrinol Metab (2008) 295:E851–8 10.1152/ajpendo.90545.2008 18664593

[23] Durstine JL Grandjean PW Davis PG Ferguson MA Alderson NL DuBose KD Blood lipid and lipoprotein adaptations to exercise: a quantitative analysis. Sports Med (2001) 31:1033–62 10.2165/00007256-200131150-00002 11735685

[24] Henderson GC Krauss RM Fattor JA Faghihnia N Luke-Zeitoun M Brooks GA Plasma triglyceride concentrations are rapidly reduced following individual bouts of endurance exercise in women. Eur J Appl Physiol (2010) 109:721–30 10.1007/s00421-010-1409-7 20217117 PMC2883923

[25] Bellou E Siopi A Galani M Maraki M Tsekouras YE Panagiotakos DB Acute effects of exercise and calorie restriction on triglyceride metabolism in women. Med Sci Sports Exerc (2013) 45:455–61 10.1249/MSS.0b013e318278183e 23073216 PMC3660976

[26] Carter SL Rennie C Tarnopolsky MA Substrate utilization during endurance exercise in men and women after endurance training. Am J Physiol Endocrinol Metab (2001) 280:E898–907 11350771 10.1152/ajpendo.2001.280.6.E898

[27] Devries MC Hamadeh MJ Phillips SM Tarnopolsky MA Menstrual cycle phase and sex influence muscle glycogen utilization and glucose turnover during moderate-intensity endurance exercise. Am J Physiol Regul Integr Comp Physiol (2006) 291:R1120–8 10.1152/ajpregu.00700.2005 16690766

[28] Devries MC Lowther SA Glover AW Hamadeh MJ Tarnopolsky MA IMCL area density, but not IMCL utilization, is higher in women during moderate-intensity endurance exercise, compared with men. Am J Physiol Regul Integr Comp Physiol (2007) 293:R2336–42 10.1152/ajpregu.00510.2007 17913867

[29] Friedlander AL Casazza GA Horning MA Buddinger TF Brooks GA Effects of exercise intensity and training on lipid metabolism in young women. Am J Physiol (1998) 275:E853–63 9815006 10.1152/ajpendo.1998.275.5.E853

[30] Friedlander AL Casazza GA Horning MA Usaj A Brooks GA Endurance training increases fatty acid turnover, but not fat oxidation, in young men. J Appl Physiol (1999) 86:2097–105 10368378 10.1152/jappl.1999.86.6.2097

[31] Henderson GC Fattor JA Horning MA Faghihnia N Luke-Zeitoun M Brooks GA Retention of intravenously infused [13C]bicarbonate is transiently increased during recovery from hard exercise. J Appl Physiol (2007) 103:1604–12 10.1152/japplphysiol.00309.2007 17702837

[32] Horton TJ Pagliassotti MJ Hobbs K Hill JO Fuel metabolism in men and women during and after long-duration exercise. J Appl Physiol (1998) 85:1823–32 9804587 10.1152/jappl.1998.85.5.1823

[33] Phillips SM Atkinson SA Tarnopolsky MA MacDougall JD Gender differences in leucine kinetics and nitrogen balance in endurance athletes. J Appl Physiol (1993) 75:2134–41 8307870 10.1152/jappl.1993.75.5.2134

[34] Tarnopolsky LJ MacDougall JD Atkinson SA Tarnopolsky MA Sutton JR Gender differences in substrate for endurance exercise. J Appl Physiol (1990) 68:302–8 2179207 10.1152/jappl.1990.68.1.302

[35] Tarnopolsky MA Rennie CD Robertshaw HA Fedak-Tarnopolsky SN Devries MC Hamadeh MJ Influence of endurance exercise training and sex on intramyocellular lipid and mitochondrial ultrastructure, substrate use, and mitochondrial enzyme activity. Am J Physiol Regul Integr Comp Physiol (2007) 292:R1271–8 10.1152/ajpregu.00472.2006 17095651

[36] Lamont LS McCullough AJ Kalhan SC Gender differences in leucine, but not lysine, kinetics. J Appl Physiol (1985) 91(2001):357–62 11408452 10.1152/jappl.2001.91.1.357

[37] Lamont LS McCullough AJ Kalhan SC Gender differences in the regulation of amino acid metabolism. J Appl Physiol (1985) 95(2003):1259–65 12807899 10.1152/japplphysiol.01028.2002

[38] McKenzie S Phillips SM Carter SL Lowther S Gibala MJ Tarnopolsky MA Endurance exercise training attenuates leucine oxidation and BCOAD activation during exercise in humans. Am J Physiol Endocrinol Metab (2000) 278:E580–7 10751189 10.1152/ajpendo.2000.278.4.E580

[39] Gaesser GA Brooks GA Metabolic bases of excess post-exercise oxygen consumption: a review. Med Sci Sports Exerc (1984) 16:29–43 10.1249/00005768-198401000-00008 6369064

[40] Brooks GA Fahey TD Baldwin KM Exercise Physiology: Human Bioenergetics and its Applications. Boston, MA: McGraw-Hill (2005).

[41] Henderson GC Fattor JA Horning MA Faghihnia N Johnson ML Luke-Zeitoun M Glucoregulation is more precise in women than in men during postexercise recovery. Am J Clin Nutr (2008) 87:1686–94 18541557 10.1093/ajcn/87.6.1686

[42] Brooks GA Importance of the “crossover” concept in exercise metabolism. Clin Exp Pharmacol Physiol (1997) 24:889–95 10.1111/j.1440-1681.1997.tb02712.x 9363377

[43] Brooks GA Mercier J Balance of carbohydrate and lipid utilization during exercise: the “crossover” concept. J Appl Physiol (1994) 76:2253–61 7928844 10.1152/jappl.1994.76.6.2253

[44] Brooks GA Trimmer JK Glucose kinetics during high-intensity exercise and the crossover concept. J Appl Physiol (1996) 80:1073–5 8964726 10.1152/jappl.1996.80.3.1073

[45] Bahr R Sejersted OM Effect of feeding and fasting on excess postexercise oxygen consumption. J Appl Physiol (1991) 71:2088–93 1778897 10.1152/jappl.1991.71.6.2088

[46] Gill JM Frayn KN Wootton SA Miller GJ Hardman AE Effects of prior moderate exercise on exogenous and endogenous lipid metabolism and plasma factor VII activity. Clin Sci (Lond) (2001) 100:517–27 10.1042/CS20000258 11294692

[47] Thomas TR Londeree BR Lawson DA Prolonged recovery from eccentric versus concentric exercise. Can J Appl Physiol (1994) 19:441–50 10.1139/h94-036 7849660

[48] Mott DM Pratley RE Bogardus C Postabsorptive respiratory quotient and insulin-stimulated glucose storage rate in nondiabetic pima Indians are related to glycogen synthase fractional activity in cultured myoblasts. J Clin Invest (1998) 101:2251–6 10.1172/JCI1778 9593781 PMC508813

[49] Coppack SW Persson M Judd RL Miles JM Glycerol and nonesterified fatty acid metabolism in human muscle and adipose tissue in vivo. Am J Physiol (1999) 276:E233–40 9950781 10.1152/ajpendo.1999.276.2.E233

[50] Jensen MD Regional glycerol and free fatty acid metabolism before and after meal ingestion. Am J Physiol (1999) 276:E863–9 10329980 10.1152/ajpendo.1999.276.5.E863

[51] Romijn JA Coyle EF Sidossis LS Gastaldelli A Horowitz JF Endert E Regulation of endogenous fat and carbohydrate metabolism in relation to exercise intensity and duration. Am J Physiol (1993) 265:E380–91 8214047 10.1152/ajpendo.1993.265.3.E380

[52] Henderson GC Kinetic measurement techniques in the evaluation of lipid metabolism. Curr Drug Discov Technol (2013) 10:209–23 10.2174/1570163811310030004 23521572

[53] Wolfe RR Peters EJ Klein S Holland OB Rosenblatt J Gary H Jr Effect of short-term fasting on lipolytic responsiveness in normal and obese human subjects. Am J Physiol (1987) 252:E189–96 3548419 10.1152/ajpendo.1987.252.2.E189

[54] Carlson MG Snead WL Campbell PJ Fuel and energy metabolism in fasting humans. Am J Clin Nutr (1994) 60:29–36 8017334 10.1093/ajcn/60.1.29

[55] Klein S Peters EJ Holland OB Wolfe RR Effect of short- and long-term B-adrenergic blockade on lipolysis during fasting in humans. Am J Physiol Endocrinol Metab (1989) 257:E65–73 2546438 10.1152/ajpendo.1989.257.1.E65

[56] Webber J Taylor J Greathead H Dawson J Buttery PJ Macdonald IA Effects of fasting on fatty acid kinetics and on the cardiovascular, thermogenic and metabolic responses to the glucose clamp. Clin Sci (1994) 87:697–706 7874862 10.1042/cs0870697

[57] Romijn JA Endert E Saurwein HP Glucose and fat metabolism during short-term starvation in cirrhosis. Gastroenterology (1991) 100:731–7 1993494 10.1016/0016-5085(91)80018-5

[58] Mittendorfer B Horowitz JF Klein S Gender differences in lipid and glucose kinetics during short-term fasting. Am J Physiol Endocrinol Metab (2001) 281:E1333–9 11701450 10.1152/ajpendo.2001.281.6.E1333

[59] Friedlander AL Casazza GA Horning MA Budinger TF Brooks GA Effects of exercise intensity and training on lipid metabolism in young women. Am J Physiol (1998) 275:E853–63 9815006 10.1152/ajpendo.1998.275.5.E853

[60] Jacobs KA Casazza GA Suh S-H Horning MA Brooks GA Fatty acid reesterification but not oxidation is increased by oral contraceptive use in women. J Appl Physiol (2005) 98:1720–32 10.1152/japplphysiol.00685.2004 15618322

[61] Magkos F Patterson BW Mohammed BS Mittendorfer B A single 1-h bout of evening exercise increases basal FFA flux without affecting VLDL-triglyceride and VLDL-apolipoprotein B-100 kinetics in untrained lean men. Am J Physiol Endocrinol Metab (2007) 292:E1568–74 10.1152/ajpendo.00636.2006 17264219

[62] Gill JM Al-Mamari A Ferrell WR Cleland SJ Perry CG Sattar N Effect of prior moderate exercise on postprandial metabolism in men with type 2 diabetes: heterogeneity of responses. Atherosclerosis (2007) 194:134–43 10.1016/j.atherosclerosis.2006.10.007 17092507

[63] Heath RB Karpe F Milne RW Burdge GC Wootton SA Frayn KN Dietary fatty acids make a rapid and substantial contribution to VLDL-triacylglycerol in the fed state. Am J Physiol Endocrinol Metab (2007) 292:E732–9 10.1152/ajpendo.00409.2006 17090753

[64] Knuth ND Horowitz JF The elevation of ingested lipids within plasma chylomicrons is prolonged in men compared with women. J Nutr (2006) 136:1498–503 16702311 10.1093/jn/136.6.1498

[65] Puga GM Meyer C Mandarino LJ Katsanos CS Postprandial spillover of dietary lipid into plasma is increased with moderate amounts of ingested fat and is inversely related to adiposity in healthy older men. J Nutr (2012) 142:1806–11 10.3945/jn.112.162008 22955513 PMC3442794

[66] Bansal S Buring JE Rifai N Mora S Sacks FM Ridker PM Fasting compared with nonfasting triglycerides and risk of cardiovascular events in women. JAMA (2007) 298:309–16 10.1001/jama.298.3.309 17635891

[67] Freiberg JJ Tybjaerg-Hansen A Jensen JS Nordestgaard BG Nonfasting triglycerides and risk of ischemic stroke in the general population. JAMA (2008) 300:2142–52 10.1001/jama.2008.621 19001625

[68] Langsted A Freiberg JJ Nordestgaard BG Fasting and nonfasting lipid levels: influence of normal food intake on lipids, lipoproteins, apolipoproteins, and cardiovascular risk prediction. Circulation (2008) 118:2047–56 10.1161/CIRCULATIONAHA.108.804146 18955664

[69] Nordestgaard BG Benn M Schnohr P Tybjaerg-Hansen A Nonfasting triglycerides and risk of myocardial infarction, ischemic heart disease, and death in men and women. JAMA (2007) 298:299–308 10.1001/jama.298.3.299 17635890

[70] Stampfer MJ Krauss RM Ma J Blanche PJ Holl LG Sacks FM A prospective study of triglyceride level, low-density lipoprotein particle diameter, and risk of myocardial infarction. JAMA (1996) 276:882–8 10.1001/jama.1996.03540110036029 8782637

[71] Tsetsonis NV Hardman AE Mastana SS Acute effects of exercise on postprandial lipemia: a comparative study in trained and untrained middle-aged women. Am J Clin Nutr (1997) 65:525–33 9022540 10.1093/ajcn/65.2.525

[72] Aldred HE Perry IC Hardman AE The effect of a single bout of brisk walking on postprandial lipemia in normolipidemic young adults. Metabolism (1994) 43:836–41 10.1016/0026-0495(94)90263-1 8028506

[73] Ferreira AP Ferreira CB Souza VC Cordova CO Silva GC Nobrega Ode T The influence of intense intermittent versus moderate continuous exercise on postprandial lipemia. Clinics (Sao Paulo) (2011) 66:535–41 10.1590/S1807-59322011000400003 21655743 PMC3093782

[74] Gill JM Al-Mamari A Ferrell WR Cleland SJ Packard CJ Sattar N Effects of prior moderate exercise on postprandial metabolism and vascular function in lean and centrally obese men. J Am Coll Cardiol (2004) 44:2375–82 10.1016/j.jacc.2004.09.035 15607401

[75] Gill JM Herd SL Hardman AE Moderate exercise and post-prandial metabolism: issues of dose-response. J Sports Sci (2002) 20:961–7 10.1080/026404102321011715 12477005

[76] Herd SL Kiens B Boobis LH Hardman AE Moderate exercise, postprandial lipemia, and skeletal muscle lipoprotein lipase activity. Metabolism (2001) 50:756–62 10.1053/meta.2001.24199 11436177

[77] Maraki M Magkos F Christodoulou N Aggelopoulou N Skenderi KP Panagiotakos D One day of moderate energy deficit reduces fasting and postprandial triacylglycerolemia in women: the role of calorie restriction and exercise. Clin Nutr (2010) 29:459–63 10.1016/j.clnu.2009.10.007 19926367

[78] Singhal A Trilk JL Jenkins NT Bigelman KA Cureton KJ Effect of intensity of resistance exercise on postprandial lipemia. J Appl Physiol (2009) 106:823–9 10.1152/japplphysiol.90726.2008 19150861

[79] Tsetsonis NV Hardman AE Reduction in postprandial lipemia after walking: influence of exercise intensity. Med Sci Sports Exerc (1996) 28:1235–42 10.1097/00005768-199610000-00005 8897379

[80] Zafeiridis A Goloi E Petridou A Dipla K Mougios V Kellis S Effects of low- and high-volume resistance exercise on postprandial lipaemia. Br J Nutr (2007) 97:471–7 10.1017/S0007114507336787 17313708

[81] Zhang JQ Ji LL Fogt DL Fretwell VS Effect of exercise duration on postprandial hypertriglyceridemia in men with metabolic syndrome. J Appl Physiol (2007) 103:1339–45 10.1152/japplphysiol.00181.2007 17641215

[82] Zhang JQ Ji LL Nunez G Feathers S Hart CL Yao WX Effect of exercise timing on postprandial lipemia in hypertriglyceridemic men. Can J Appl Physiol (2004) 29:590–603 10.1139/h04-038 15507695

[83] Zotou E Magkos F Koutsari C Fragopoulou E Nomikos T Sidossis LS Acute resistance exercise attenuates fasting and postprandial triglyceridemia in women by reducing triglyceride concentrations in triglyceride-rich lipoproteins. Eur J Appl Physiol (2010) 110:869–74 10.1007/s00421-010-1561-0 20607278

[84] Katsanos CS Moffatt RJ Acute effects of premeal versus postmeal exercise on postprandial hypertriglyceridemia. Clin J Sport Med (2004) 14:33–9 10.1097/00042752-200401000-00006 14712164

[85] Uranga AP Levine J Jensen M Isotope tracer measures of meal fatty acid metabolism: reproducibility and effects of the menstrual cycle. Am J Physiol Endocrinol Metab (2005) 288:E547–55 10.1152/ajpendo.00340.2004 15507534

[86] Matthan NR Jalbert SM Barrett PH Dolnikowski GG Schaefer EJ Lichtenstein AH Gender-specific differences in the kinetics of nonfasting TRL, IDL, and LDL apolipoprotein B-100 in men and premenopausal women. Arterioscler Thromb Vasc Biol (2008) 28:1838–43 10.1161/ATVBAHA.108.163931 18658047 PMC2872098

[87] Nguyen TT Hernandez Mijares A Johnson CM Jensen MD Postprandial leg and splanchnic fatty acid metabolism in nonobese men and women. Am J Physiol (1996) 271:E965–72 8997213 10.1152/ajpendo.1996.271.6.E965

[88] Dallongeville J Gruson E Dallinga-Thie G Pigeyre M Gomila S Romon M Effect of weight loss on the postprandial response to high-fat and high-carbohydrate meals in obese women. Eur J Clin Nutr (2007) 61:711–8 10.1038/sj.ejcn.1602603 17228347

[89] Lewis GF O’Meara NM Soltys PA Blackman JD Iverius PH Druetzler AF Postprandial lipoprotein metabolism in normal and obese subjects: comparison after the vitamin A fat-loading test. J Clin Endocrinol Metab (1990) 71:1041–50 10.1210/jcem-71-4-1041 2401706

[90] van Beek AP de Ruijter-Heijstek FC Erkelens DW de Bruin TW Menopause is associated with reduced protection from postprandial lipemia. Arterioscler Thromb Vasc Biol (1999) 19:2737–41 10.1161/01.ATV.19.11.2737 10559019

[91] Mittendorfer B Patterson BW Klein S Effect of sex and obesity on basal VLDL-triacylglycerol kinetics. Am J Clin Nutr (2003) 77:573–9 12600845 10.1093/ajcn/77.3.573

[92] Magkos F Patterson BW Mohammed BS Klein S Mittendorfer B Women produce fewer but triglyceride-richer very low-density lipoproteins than men. J Clin Endocrinol Metab (2007) 92:1311–8 10.1210/jc.2006-2215 17264179

[93] Tsekouras YE Yanni AE Bougatsas D Kavouras SA Sidossis LS A single bout of brisk walking increases basal very low-density lipoprotein triacylglycerol clearance in young men. Metabolism (2007) 56:1037–43 10.1016/j.metabol.2007.03.012 17618947

[94] Bellou E Magkos F Kouka T Bouchalaki E Sklaveniti D Maraki M Effect of high-intensity interval exercise on basal triglyceride metabolism in non-obese men. Appl Physiol Nutr Metab (2013) 38:823–9 10.1139/apnm-2012-0468 23855269

[95] Tsekouras YE Magkos F Prentzas KI Basioukas KN Matsama SG Yanni AE A single bout of whole-body resistance exercise augments basal VLDL-triacylglycerol removal from plasma in healthy untrained men. Clin Sci (Lond) (2009) 116:147–56 10.1042/CS20080078 18554182

[96] Linden MA Fletcher JA Morris EM Meers GM Kearney ML Crissey JM Combining metformin and aerobic exercise training in the treatment of type 2 diabetes and NAFLD in OLETF rats. Am J Physiol Endocrinol Metab (2014) 306:E300–10 10.1152/ajpendo.00427.2013 24326426 PMC3920010

[97] Linden MA Meers GM Ruebel ML Jenkins NT Booth FW Laughlin MH Hepatic steatosis development with four weeks of physical inactivity in previously active, hyperphagic OLETF rats. Am J Physiol Regul Integr Comp Physiol (2013) 304:R763–71 10.1152/ajpregu.00537.2012 23467323 PMC3652080

[98] Rector RS Uptergrove GM Morris EM Borengasser SJ Laughlin MH Booth FW Daily exercise vs. caloric restriction for prevention of nonalcoholic fatty liver disease in the OLETF rat model. Am J Physiol Gastrointest Liver Physiol (2011) 300:G874–83 10.1152/ajpgi.00510.2010 21350190 PMC3094141

[99] Thyfault JP Rector RS Uptergrove GM Borengasser SJ Morris EM Wei Y Rats selectively bred for low aerobic capacity have reduced hepatic mitochondrial oxidative capacity and susceptibility to hepatic steatosis and injury. J Physiol (2009) 587:1805–16 10.1113/jphysiol.2009.169060 19237421 PMC2683966

[100] Grove KL Fried SK Greenberg AS Xiao XQ Clegg DJ A microarray analysis of sexual dimorphism of adipose tissues in high-fat-diet-induced obese mice. Int J Obes (Lond) (2010) 34:989–1000 10.1038/ijo.2010.12 20157318 PMC3667412

[101] Garthwaite SM Cheng H Bryan JE Craig BW Holloszy JO Ageing, exercise and food restriction: effects on body composition. Mech Ageing Dev (1986) 36:187–96 10.1016/0047-6374(86)90019-9 3784631

[102] Holloszy JO Exercise increases average longevity of female rats despite increased food intake and no growth retardation. J Gerontol (1993) 48:B97–100 10.1093/geronj/48.3.B97 8482812

[103] Holloszy JO Smith EK Vining M Adams S Effect of voluntary exercise on longevity of rats. J Appl Physiol (1985) 59(1985):826–31 4055572 10.1152/jappl.1985.59.3.826

[104] Oscai LB Mole PA Krusack LM Holloszy JO Detailed body composition analysis on female rats subjected to a program of swimming. J Nutr (1973) 103:412–8 4688153 10.1093/jn/103.3.412

[105] Slentz CA Holloszy JO Body composition of physically inactive and active 25-month-old female rats. Mech Ageing Dev (1993) 69:161–6 10.1016/0047-6374(93)90020-R 8412367

[106] Ballor DL Keesey RE A meta-analysis of the factors affecting exercise-induced changes in body mass, fat mass, and fat-free mass in males and females. Int J Obes (1991) 15:717–26 1838100

[107] Donnelly JE Smith BK Is exercise effective for weight loss with ad libitum diet? Energy balance, compensation, and gender differences. Exerc Sport Sci Rev (2005) 33:169–74 10.1097/00003677-200510000-00004 16239833

[108] Brown LM Clegg DJ Central effects of estradiol in the regulation of food intake, body weight, and adiposity. J Steroid Biochem Mol Biol (2010) 122:65–73 10.1016/j.jsbmb.2009.12.005 20035866 PMC2889220

[109] Hill JO Talano CM Nickel M DiGirolamo M Energy utilization in food-restricted female rats. J Nutr (1986) 116:2000–12 3772527 10.1093/jn/116.10.2000

[110] Gambacciani M Ciaponi M Cappagli B Benussi C De Simone L Genazzani AR Climacteric modifications in body weight and fat tissue distribution. Climacteric (1999) 2:37–44 10.3109/13697139909025561 11910677

[111] Ho SC Wu S Chan SG Sham A Menopausal transition and changes of body composition: a prospective study in Chinese perimenopausal women. Int J Obes (Lond) (2010) 34:1265–74 10.1038/ijo.2010.33 20195288

[112] Lovejoy JC Champagne CM de Jonge L Xie H Smith SR Increased visceral fat and decreased energy expenditure during the menopausal transition. Int J Obes (Lond) (2008) 32:949–58 10.1038/ijo.2008.25 18332882 PMC2748330

