# Corrigendum: Sexual Dimorphism in the Effects of Exercise on Metabolism of Lipids to Support Resting Metabolism

**DOI:** 10.3389/fendo.2014.00200

**Published:** 2014-11-27

**Authors:** Gregory C. Henderson

**Affiliations:** ^1^Newomics Inc., Emeryville, CA, USA

**Keywords:** gender, fat metabolism, Lipids, physical activity, metabolic rate

**Amendment**

Current address of the author is Newomics Inc., Emeryville, CA.

## Conflict of Interest Statement

The author declares that the research was conducted in the absence of any commercial or financial relationships that could be construed as a potential conflict of interest.

